# Country-Wide HCV Elimination Strategies Need to Reach Older Patients in the General Population: The Italian Experience

**DOI:** 10.3390/v15112199

**Published:** 2023-10-31

**Authors:** Pietro Torre, Roberta Coppola, Mario Masarone, Marcello Persico

**Affiliations:** Internal Medicine and Hepatology Unit, Department of Medicine, Surgery and Dentistry, Scuola Medica Salernitana, University of Salerno, Largo Città d’Ippocrate, 84131 Salerno, Italy; ptorre@unisa.it (P.T.); rcoppola@unisa.it (R.C.); mmasarone@unisa.it (M.M.)

**Keywords:** hepatitis C, HCV screening, linkage-to-care, viral hepatitis elimination

## Abstract

HCV infection is still a major burden worldwide, and most countries are not on track to meet the WHO 2030 elimination goal. The current challenge is to identify individuals to be treated. In this study, we will describe the trend of new DAA prescriptions and the changes over time in terms of the characteristics of patients starting antiviral therapy in our unit. Data of 1646 hepatitis C patients who started therapy during the period of 2015–2022 regarding annual number of prescriptions, age, gender, nationality, HCV genotype, provenance, and liver disease severity were analyzed. We observed a peak in the number of new prescriptions in 2018 and a downward trend starting in 2019. Patients from the general population, centers for addictions, and prison differed significantly. The mean age in the general population remained above 60 years, the percentage of patients from centers for addictions and prison increased and, after 2016, there was no significant change in the percentage of patients with F3–F4 fibrosis. As HCV screening and linkage-to-care pathways seem to be already well implemented and successful in centers for addictions and in prisons, efforts need to be focused on those of older age in the general population. To carry this out, the more structured involvement of different health professionals must be figured out.

## 1. Introduction

According to the World Health Organization (WHO), chronic hepatitis C virus (HCV) infection affects approximately 58 million people worldwide [1]. In Italy, which was earlier considered a country with a high burden of HCV infection, and with an increasing trend towards older ages and the southern regions, the Polaris Observatory recently estimated a prevalence of around 1% [2,3,4,5,6,7,8]. Although this infection is still a significant cause of liver-related morbidity and mortality, nowadays, it can be easily cured with direct-acting antivirals (DAAs) and, according to current guidelines, all patients with HCV infection are recommended to start antiviral therapy, regardless of the severity of liver disease, with a few exceptions. In fact, these drugs can eliminate the infection with a very high success rate and in a short time [9,10]. Italy has been a leading country for the number of DAA prescriptions since their advent; Campania, a region with a high prevalence of infection, has initiated the largest number of treatments by number of inhabitants and is in second place, after Lombardy, by absolute number (unpublished data from the Italian Drug Agency—*Agenzia Italiana del Farmaco*, AIFA) [7]. Supported by advancements in treatment, in 2016, the WHO adopted the goal of eliminating viral hepatitis, mostly related to hepatitis B and C, as a major public health threat by 2030. In this regard, the use of specific impact (80% reduction in incidence, referring to HCV, and 65% reduction in mortality) and programmatic (≥90% of people diagnosed, and ≥80% of those diagnosed treated) targets was suggested [11,12]. Anyway, just a few years from 2030, there is still a lot of work to be conducted globally. Current efforts are focused on diagnosing and linking to care as many individuals as possible, and monitoring the progress towards elimination is an additional complex challenge to be carried out [13,14]. To make HCV elimination less daunting, the European Association for the Study of the Liver (EASL) proposed the “micro-elimination strategy”, aimed at simplifying attainment of identified targets by focusing on specific groups of people, such as prison inmates, intravenous drug users, and migrants from high-prevalence areas [15]. Although our country was previously listed among those on track to achieve HCV elimination by 2030, it has subsequently gone backwards [2,7,16]. In this retrospective, consecutive study, we will describe the trend of new DAA prescriptions and the changes we observed in our unit, in a timeframe from the beginning of DAA prescription to recent day (2015–2022), regarding the characteristics of patients who started treatment, with a prominent focus on age, source population, and proportion of those who had more severe liver disease, with the aim of depicting how the management of hepatitis C has progressed, which, in turn, can be useful in defining how to best continue in the years to come.

## 2. Patients and Methods

We analyzed the data of 1646 HCV patients, consecutively treated with DAAs in our unit from March 2015 to December 2022, regarding the yearly number of prescriptions, the patient age at the time of prescription, gender, nationality, HCV genotype, provenience (general population, centers for addictions (*Servizi per le Dipendenze*—SerD), or prison), and liver disease severity. Liver disease severity was assessed before the start of treatment through clinical evaluation, blood tests, ultrasound and liver stiffness measurement (LSM) by transient elastography (TE) with FibroScan^®^ (Echosens, Paris, France), performed according to quality criteria [17,18], and by using the following cutoffs to identify patients with more severe liver disease: ≥10 kPa for advanced fibrosis (METAVIR score F3) and ≥13 kPa for cirrhosis (METAVIR score F4) [10,19]. After assessment of normality, one-way ANOVA or Kruskal–Wallis tests were used to compare continuous variables. The Chi-square test was used to compare the proportions. A *p*-value < 0.05 was defined as statistically significant. Statistical analysis was performed with GraphPad Prism 9.5.0. The retrospective data analysis for this study was submitted to the Ethics Committee “Comitato etico Campania Sud”. Collection of informed consent was not applicable for this study.

## 3. Results

Of the total number of patients who were prescribed DAA therapy between 2015 and 2022, the mean age was 60.84 years, 963 patients (58.51%) were male and 683 (41.49%) were female, 52 patients (3.16%) were of foreign nationality, the most frequent HCV genotype was 1b (39.85%), and 876 patients (53.22%) had F3 or F4 fibrosis. The number of prescriptions and patient characteristics according to the different source populations are summarized in Table 1.

**Figure 1:**
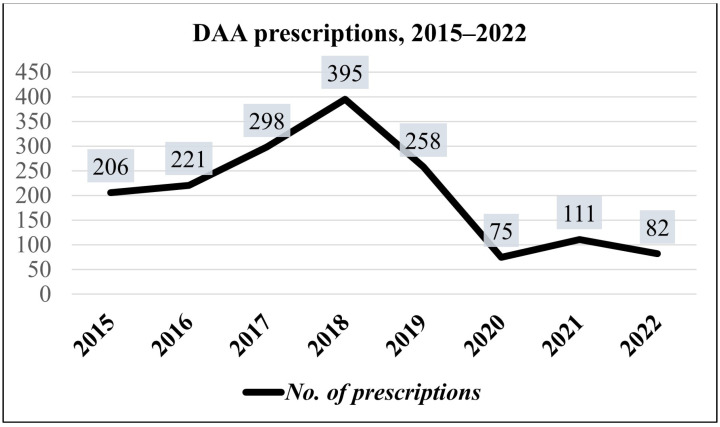
Number of DAA prescriptions from year to year (2015–2022).

Figure 1 illustrates the change in the number of new prescriptions in these years. A peak in prescriptions was reached in 2018; then, starting in 2019, there was a decline. In 2020, the lowest number of prescriptions was registered.

**Figure 2:**
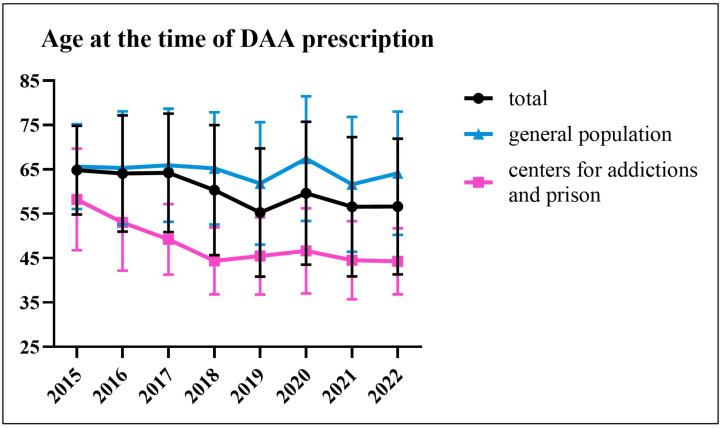
Mean age and standard deviation (SD) of patients starting antiviral therapy for HCV in each year (2015–2022).

As shown in Figure 2, the mean age of patients starting antiviral therapy changed over the years (total *p* < 0.0001, general population *p* = 0.0107, and centers for addictions and prison *p* < 0.0001).

**Figure 3:**
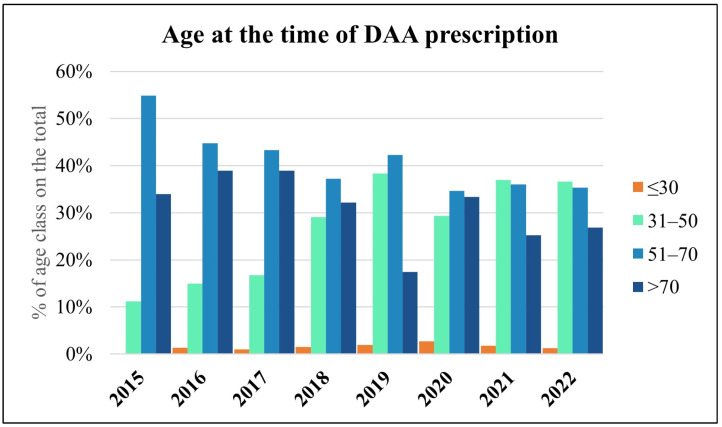
Total number of patients, expressed as percentage of age class, starting antiviral therapy for HCV in each year (2015–2022).

**Figure 4:**
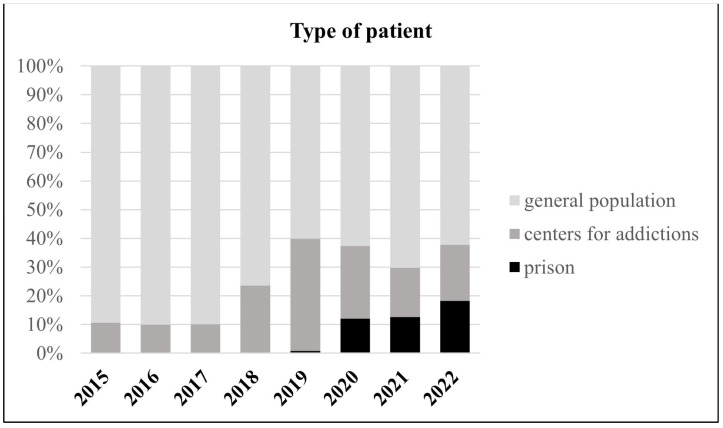
Percentage of patient type (general population, centers for addictions, or prison) starting antiviral therapy for HCV in each year (2015–2022).

Age-class analysis of the total number of patients (Figure 3) shows an increasing trend for people aged between 31 and 50, in parallel with the increase in the proportion of subjects from centers for addictions, with an average age of 47.11 years, and prison inmates with an average age of 42.78 years (Figure 4).

**Figure 5:**
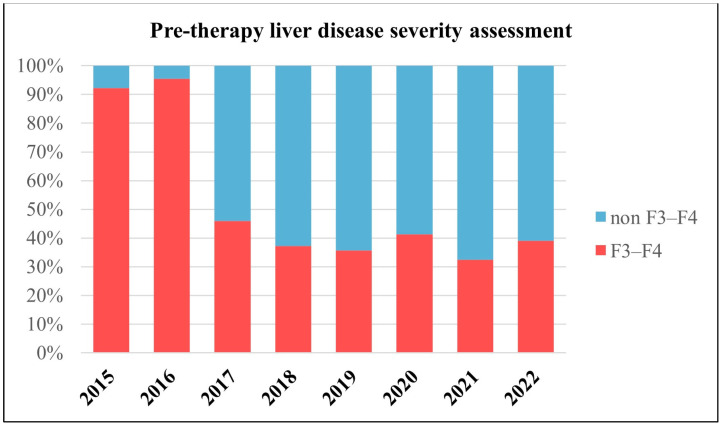
Percentage of patients with F3–F4 fibrosis starting antiviral therapy for HCV in each year (2015–2022).

When only the general population is considered, the mean age exceeded 60 in each year (Figure 2). The high percentage of F3–F4 patients in the total number of patients in 2015 and 2016 (Figure 5) was attributable to the regulations in force in those years. There was no significant difference in the percentage of F3–F4 patients starting antiviral therapy between 2017 and 2022 (*p* = ns).

## 4. Discussion

In Italy, a peculiar policy of access to DAAs was adopted in the first two years of their availability (2015–2016), and the treatment was possible for F3–F4 patients, subjects with HCV-related disorders (non-Hodgkin lymphoma and mixed cryoglobulinemia), some patients with hepatocellular carcinoma (HCC), and in the context of transplants. Then, in March 2017, restrictions on access to DAAs were removed by the Italian Drug Agency, and therapy was made universally accessible, regardless of the stage of fibrosis and the presence of comorbidities. Also because of these new policies, our country has been listed among those on track to reach HCV elimination by 2030 [16,20,21,22]. However, it has been estimated that without an increase in screening activities, the pool of patients to be treated could run out by 2025, leaving a large number of people without diagnosis and treatment [23]. Afterwards, national data show that there was a consistent reduction in the number of DAA prescriptions starting from the year 2019 [20,24,25], before the outbreak of the SARS-CoV-2 pandemic. The latter nonetheless had a major negative impact on the management of viral hepatitis. In fact, the results of a survey by the Italian Association for the Study of the Liver (*Associazione Italiana per lo Studio del Fegato*—AISF), in which 194 members were asked about the impact of COVID-19 on hepatological activities in Italy, documented that the start of any antiviral therapy was completely interrupted in 23.7% of the centers, and that only in 17.2% of the cases were there no changes [26,27]. Due to those events, Italy is no longer considered on track to meet the WHO elimination targets for HCV, except for the 65% reduction in mortality [2,7]. It was estimated that the reduction in the number of patients treated in Italy, exacerbated by the pandemic, will have consequences in the form of a high burden of liver-related events (decompensation and HCC) and death caused by HCV even beyond 2030 unless screening and treatment rates increase considerably [28].

In line with national data, in our unit we observed a decline in new DAA prescriptions starting in 2019, with a further reduction occurring in 2020 during the first two SARS-CoV-2 pandemic outbreaks. Our data also show that, after the end of the lockdown policies, the previous treatment rates were not fully restored. After an initial increase in treatments during 2021 (compared to 2020), a new decrease occurred in 2022, in line with what was observed throughout the Campania region (unpublished AIFA data). The decline in the number of prescriptions since 2019 in Italy was mostly attributed to the depletion of patients with an active infection already linked to care, awaiting treatment. This phenomenon is considered a cause of the recent decline in treatment rates worldwide [8,24].

To re-establish adequate treatment rates, it is necessary not only to carry out screening activities, but also to ensure that the identified infected subjects are treated. In a Markov model study, published by Kondili et al., various screening strategies to be implemented in our country were evaluated. The most advantageous turned out to be a “gradual” screening, starting from young populations and then extending to older ones [29]. Accordingly, the Italian government allocated 71.5 million euros to carry out an experimental HCV screening in 2020–2021 in people born from 1969 to 1989 and in two high-prevalence populations (prison inmates and subjects from centers for addictions) [30]. Subsequently, the time frame for carrying out this activity was extended to December 2023, mainly due to impediments related to the pandemic [31]. Meanwhile (2021–2022), as was previously suggested and recommended [7,32,33], the pandemic itself was exploited to the advantage of HCV testing, that is to say SARS-CoV-2 testing or vaccination were leveraged for a concomitant HCV screening in the general population. Programs of this type were conducted in northern and southern Italy and documented a lower-than-expected prevalence of infection in the target age group [34,35,36,37].

The present study underscores the importance of making a distinction between the general population and special populations. In fact, these categories were substantially different in terms of age (which in the general population was higher and remained steadily above 60 over time), gender, HCV genotype, and percentage of those with F3–F4 fibrosis. In agreement with what is known from the literature, in our study, HCV-genotype 1b was the most frequent in the general population, while HCV-genotype 3 was the most frequent in people from centers for addictions and in prison, for whom intravenous drug use is a leading risk factor for infection [38,39,40,41,42]. HCV-genotype 3 is responsible for the largest number of infections in Asian countries, such as India and Pakistan, and is estimated to have rapidly spread to Western countries through the practice of intravenous drug use [13,40,43,44]. Other differences between the general population and the special populations we observed in our analysis, and which confirm the results of previous studies [38,39], were a prevalence of male sex and a lower burden of F3–F4 fibrosis (with this likely being the result of the younger age) in the special populations. The international hepatological community previously has indicated to focus a dedicated HCV screening in at-risk reservoirs, such as drug users and prison inmates, and these categories were also among the targets of the micro-elimination strategy [15,45]. This is well represented by the data reported here, showing that, in our unit, screening and linkage-to-care for the latter categories has been consolidated over the years through relationships established with health professionals operating in the respective facilities. Conversely, after universal access to DAAs was achieved, an HCV-screening plan dedicated to the general population was not established; therefore, the greatest challenge has consisted of identifying infected subjects in this setting. In this regard, recent studies conducted in the Campania region showed that the hospital can be a strategic site to carry out case-finding and linkage-to-care activities [3,21,46]. In the hospital, in fact, a high prevalence of infection was found, and patients could start therapy either during hospitalization or could be contacted for evaluation and therapy after discharge. Also in these cases, the prevalence of infection was age related, with it being lower in younger cohorts [3,21]. In a recent multicenter study conducted in Lombardy on over 120,000 subjects who underwent HCV screening in hospitals or blood collection centers, a prevalence of active infection of 0.10% was found in the 1969–1989 cohort [47]. To date, despite these results and despite having been recommended, an extension of the free screening to people from the general population born before 1969 has not yet been decreed [48,49]. General practice and pharmacies are additional contexts where it is worth investing in HCV screening and linkage-to-care in the general population, but in these cases, there is also a need for an increase in knowledge of the disease and current therapeutic strategies, and in coordination with liver specialists [7,48,50,51].

Lastly, we observed that, after 2016, there was no significant difference in the percentage of F3–F4 patients who started therapy. This finding can be considered a proof of the current incomplete efficiency of the HCV cascade of care, and shines a light on the issue, also raised in the recent studies carried out in the Campania region [3,21], of the likelihood of a high number of people with HCV-related advanced liver disease still untreated [52]. Similarly, nationwide data have shown that after the initial phase in which most of those treated with DAAs were people with advanced liver fibrosis or cirrhosis, the percentage of these subjects decreased but remained stable (around 30%) [24,53]. It is imperative to promptly identify and treat those patients because the achievement of SVR in such populations is proven to reduce the occurrence of liver-related events, including HCC. However, since the risk is not entirely eliminated, post-SVR surveillance is suggested [10,54,55,56].

Potential limitations of our study are that our analysis included neither an evaluation of SVR rates nor of the presence of cofactors for liver disease progression, such as concomitant HBV infection, unsafe alcohol consumption, or metabolic risk factors, and therefore, it was not possible to make a comparison based on these aspects across the three different settings and over the years. Although viral hepatitis is still the worldwide leading cause of cirrhosis, also due to the expected reduction in HCV and HBV infections, cirrhosis caused by non-alcoholic steatohepatitis (NASH) and cirrhosis due to alcohol-related liver disease (ALD) are estimated to become dominant [57,58]. In Italy, although there is not much recent data available on the burden of fibrosis caused by these latter liver diseases, a high percentage of subjects with advanced fibrosis among those affected by non-alcoholic fatty liver disease (NAFLD) in the general population and an increasing trend of ALD patients presenting with more severe liver damage were reported [59,60]. An analysis of the trends of late presentation of these liver diseases may be useful to carry out in future studies to evaluate the effectiveness of screening programs aimed at early identification.

## 5. Conclusions

Our findings on the characteristics of HCV-infected subjects who started DAA therapy in our unit over the years underscore the need for higher identification and treatment rates, mainly for older people in the general population. To bring home this ambitious goal, important actions to implement could be, in our opinion, the extension of free screening to older age cohorts and a higher commitment of other healthcare professionals, in addition to hepatologists, in the hospital, general practice, or other contexts (e.g., territorial pharmacies). These efforts could restore adequate treatment rates to meet the WHO 2030 elimination goal.

## Figures and Tables

**Table 1:** Number of prescriptions and the characteristics of the patients who started antiviral therapy in the period of 2015–2022 according to the different source populations.

	Total	General Population	Centers for Addictions	Prison	*p*
No. of prescriptions	1646	1284	322	40	/
Mean age, years (SD)	60.84 (14.28)	64.84 (12.79)	47.11 (9.466)	42.78 (7.661)	<0.0001
Male gender, No. (%)	963 (58.51%)	661 (51.48%)	266 (82.61%)	36 (90%)	<0.0001
Foreign nationality, No. (%)	52 (3.16%)	38 (2.96%)	13 (4.04%)	1 (2.5%)	0.5958
Most frequent HCV genotype (%)	1b (39.85%)	1b (47.27%)	3 (42.55%)	3 (42.5%)	/
F3–F4 fibrosis, No. (%)	876 (53.22%)	734 (57.17%)	134 (41.61%)	8 (20%)	<0.0001

## Data Availability

Additional data are available from the authors upon request.
